# Can we use LDL instead of egg yolk in BotuCrio® extender to cryopreserve sperm from the Mangalarga Marchador stallion?

**DOI:** 10.21451/1984-3143-AR2019-0039

**Published:** 2019-10-23

**Authors:** Paola Pereira das Neves Snoeck, Tainá Hortência Oliveira Pessoa, Marcelo Gonçalves Sousa Pereira, Inara Cristina Leal Bastos, Maria Isabel Vaz de Melo

**Affiliations:** 1 Universidade Estadual de Santa Cruz, Ciências Agrárias e Ambientais, Ilheus, Bahia, Brazil.; 2 Pontífica Universidade Católica de Minas Gerais, Medicina Veterinária, Belo Horizonte, Brazil.

**Keywords:** sperm, lipoproteins, amide, cryoprotection, equine

## Abstract

The objective of this study was to compare the BotuCrio^®^ extender with the Merk - egg yolk and the INRA 82 modified by the inclusion of acetamide, methyl cellulose and trehalose in substitution of glycerol for freezing equine semen. The semen was diluted after centrifugation to obtain 100 x 10^6^ of sperm/ml in: BotuCrio^®^ (control); Merk - egg yolk or INRA 82 modified (Experiment 1). The extended semen was packaged in 0.5 ml straws, cooled and frozen in a freezing machine. The control extender was superior in preserving the motility, VCL, VSL, VAP, LIN, STR and the BCF when compared to the Merk - egg yolk and INRA 82 modified (P < 0.05). The BotuCrio® preserved more effectively the equine sperm viability characteristics evaluated in Experiment 1 and was used as a control extender in Experiment 2 to test the effectiveness of using LDL in replacement of egg yolk. BotuCrio^®^ was superior to preserve progressive motility, VCL, VSL, VAP, LIN, STR and the percentage of functional integrity of sperm membranes compared to BotuCrio LDL (P < 0.05). However, both extenders preserved similarly the total motility, ALH, BCF and the structural integrity of the membranes (P > 0.05). The fertility rate after AI with frozen semen in BotuCrio LDL was 37.5%.

## Introduction

The breed Mangalarga Marchador occupies a prominent position in Brazil for the use in equestrian sports and leisure. In order to maximize the reproductive potential of stallions with high zootechnical value, the association of this breed allows the use of freezing semen in order to guarantee the preservation of sperm for an indefinite period of time. However, the large-scale use of equine frozen semen is still quite limited, because there is a breed factor related to the resistance of the gamete to the cryopreservation process. According to Alvarenga *et al.* (2005), there were semen donors from national breeds that have greater sensitivity to the cryopreservation process when compared to others that have suffered reproductive selection.

Stallion sperm is not as tolerant to cryopreservation as other species particularly if glycerol is used as the cryoprotectant (Alvarenga *et al*., 2002; 2005). Identifying alternative cryoprotectants to the glycerol to cryopreserve equine sperm has become an area of extensive investigation. Extenders based on egg yolk as the Lactose-EDTA (Merk-egg yolk) (Martin *et al*., 1979; Henry *et al*., 2002) and from milk and egg yolk as the INRA 82 (Magistrini *et al*., 1992; Vidament *et al*., 2002; Pillet *et al*., 2008; Candeias *et al*., 2012; Álvarez *et al*., 2014) containing glycerol or after replacing this by other cryoprotectors based on amides were tested to preserve sperm viability and/or the fertility during cryopreservation, in an attempt to prevent the cytotoxic effect of glycerol (Alvarenga *et al*., 2000; 2002; Henry *et al*., 2002; Gomes *et al*., 2002; Medeiros *et al*., 2002; Papa *et al*., 2002; Squires *et al*., 2004; Snoeck *et al*., 2007; 2012; Gibb et al., 2013). The amides have lower viscosity and low molecular weight which favors a greater permeability of this compound through the plasmatic membrane, causing less osmotic damages (Alvarenga *et al*., 2005; Melo *et al*., 2007) and were effective to preserve the sperms from stallions considered bad freezers (Alvarenga *et al*., 2002; Ramires Neto *et al*., 2014).

Many cryoprotectant agents of amide chemical group were tested alone or in association with glycerol (Vidament *et al*., 2002; Gomes *et al*., 2002; Medeiros *et al*., 2002). According to Gomes *et al*. (2002), the association between amides and glycerol in semen extender preserved in a better way the sperm of Mangalarga Marchador stallions than the use of glycerol alone. This discovery marked the beginning of the large-scale use of the commercial extender BotuCrio® containing an association of methylformamide and glycerol to minimize the problem of higher sensitivity to the freezing process of equine sperm.

In addition to the intracellular cryoprotectant agent, the majority of the extenders used for freezing semen contain egg yolk in its composition that is also present in BotuCrio®. The egg yolk cryoprotective effect is allocated to its low density lipoprotein fraction (LDL). The use of 8 to 10% of LDLs in the extender, extracted from egg yolk and replacing the total egg yolk fraction, has resulted in a better sperm motility and ability of fertilization of frozen bovine sperm (Moussa *et al*., 2002; Amirat *et al*., 2004; Jiang *et al*., 2007). Varela Junior *et al*. (2009) reported that the LDL can replace the egg yolk in the extender of cooling and freezing of canine semen. It was noticed a positive effect of LDL on post-thaw sperm viability of canine sperm (Neves *et al*., 2014) and ovine (Moustacas *et al*., 2011; Silva *et al*., 2014) in our laboratory. Juliani *et al*. (2004) found a slight advantage in replacing the egg yolk by 10 or 20% of LDL in the extender containing dimethylformamide to cryopreserve equine sperm. Although, Moreno *et al.* (2013) have reported that concentrations of 2% of LDL in INRA 96® extender would be enough to preserve the sperm motility. Considering these findings, the efficiency of LDL as a bioproduct in sperm cryoprotection and with a more predictable and controlled formulation than the egg yolk is an interesting alternative to make the commercial extenders chemically defined and sanitarily controlled (Pillet *et al*., 2011).

Therefore, this study had the overall objective of modifying the extenders of semen freezing aiming the increase in freezeability of Mangalarga Marchador stallions sperm and was designed in two experiments. The aim of the first experiment was to compare the BotuCrio® extender containing glycerol and methylformamide to the Merk-egg yolk and INRA 82 modified by the inclusion of acetamide, methylcellulose and trehalose in substitution of glycerol to cryopreserve equine sperm. The second experiment was designed to evaluate the effectiveness of the use of LDL in replacing the egg yolk of BotuCrio® extender on the viability and fertility of cryopreserved equine sperm.

## Materials and methods

The present work was submitted to the Animal Experimentation Ethics Committee - CEUA/UESC, State University of Santa Cruz, Ilhéus, Bahia, Brazil and had opinion approved under the N 013/11.

### Stallions, semen collection, evaluation and freezing

Mangalarga Marchador stallions in reproductive age and considered suitable for reproduction after breeding soundness evaluation were used in both experiments. The stallions were selected for their ability to produce ejaculates with more than 100 x 10^6^ sperm/ml and ≥ 60% sperm motility after a week of daily semen collection to stabilize the sperm reserve.

The semen was evaluated according to the standards of the Brazilian College of Animal Reproduction (CBRA, 2013) before freezing. The semen collected and evaluated was diluted 1:2 in milk-based extender (BotuSemen®) and centrifuged at 600 x g for 10 min. The supernatant was removed leaving a minimum of 10% of seminal plasma. The pellet with a small amount of seminal plasma was resuspended either in control or in experimental extender to obtain a 100 x 10^6^ sperm/ml and then loaded into 0.5 ml polyvinyl chloride straws (IMV-Technologies, L’Aigle, France) and sealed with polyvinyl alcohol sealing powder.

Cooling and freezing was achieved with a programmable freezer machine (TK4000® equipment, TK Tecnologia em Congelação LTDA, Uberaba, Minas Gerais, Brazil). Straws were cooled from 20.5^o^C to 5^o^C at a rate of −0.5^o^C/min, kept in equilibrium at 5^o^C for 30 min and then frozen using a freezing rate of -10^o^C/min up to -140^o^C, followed by immersion in liquid nitrogen (-196ºC).

### Experiment 1

The focus of this experiment was to compare the BotuCrio® extender with the Merk-egg yolk and the INRA 82 modified. Four Mangalarga Marchador stallions were used; two ejaculates per animal. Ejaculates were diluted, after centrifugation, either in the control extender BotuCrio® (Botupharma, Botucatu, São Paulo, Brazil), containing sugars, amino acids, glycerol and methylformamide or in experimental extenders Lactose-EDTA-egg yolk (Martin *et al*., 1979) and INRA 82 (Magistrini *et al*., 1992) both containing 5% of acetamide + 0.5% of methylcellulose + 0.165% of trehalose in substitution of glycerol (considered Merk-egg yolk modified and INRA 82 modified). The BotuCrio® extender was chosen as control because it is widely used in Brazil for freezing sperm of stallions with greater sensitivity to the process of cryopreservation.

### Experiment 2

The focus of this experiment was to test the effect of LDL in BotuCrio® extender. Single ejaculates from 14 stallions, different from those used in Experiment 1, were collected. Ejaculates were diluted, after centrifugation, either in the control extender BotuCrio® (Botupharma, Botucatu, São Paulo, Brazil), containing 10% of egg yolk and other components or in experimental BotuCrio extender with the replacement of egg yolk for 12% of LDL.

The LDL was extracted from egg yolk according to the protocol described by Moussa *et al*. (2002) and modified by Neves *et al.* (2014). The LDL was added to the extender after its extraction and a short period of frozen maintenance according to the protocol described by Snoeck *et al*. (2017).

### Thawing and assessment of sperm viability

After thawing (46ºC for 20 seconds) samples were incubated at 37^o^C and evaluated after five minutes (0h), one and two hours of incubation. Evaluated parameters were: (a) sperm motion using the Sperm Class Analyser® program (SCA®, v.5.2, Microptics S.L., Barcelona, Spain) after dilution of the samples in freezing extenders tested to reach 50 x 10^6^ sperm/ml, (b) the structural integrity of sperm membranes (CFDA/IP) and (c) the functional integrity of sperm membrane by hypoosmotic swelling test (HOST). Two straws of each stallion and of each experimental group were thawed.

The patterns used for equipment adjustment were based on the recommendations of the program SCA® for analysis sperm of equine as it follows: 25 images/second with 25 Hz; captured particle size between 4 and 75 µm/m^2^; spermatozoon considered immobile < 10 µm/s, slow < 45 µm/s, medium of 45 to 90 µm/s and fast above 90 µm/s. The following parameters measured were: Total Motility (TM, %), Progressive Motility (PM, %), Curvilinear Velocity (VCL, µm/s), Straight Linear Velocity (VSL, µm/s), Average Path Velocity (VAP, µm/s), Fast (FAST; %), Medium (MED; %), Slow (SLOW;%), Linearity (LIN, %), Straightness (STR, %), Amplitude of Lateral Head Displacement (ALH, µm), Tail Beat Frequency (BCF, Hz) and hyperactive (%). Sperm motion parameters were evaluated five minutes after thawing. The motility was also evaluated after one and two hours of incubation at 37^o^C.

The structural integrity of the plasma and acrosomal membranes was evaluated using a fluorescent microscope (400X; Olympus® CX 51) after staining the sperm with the fluorescent dyes carboxyfluorescein diacetate (CFDA) and propidium iodide (PI) according to the method of Harrison and Vickers (1990). Staining with CFDA was assessed using the standard fluorescein filter set, while staining with PI was assessed using the standard rhodamine filter set. There were analyzed 200 sperms per sample. The functional integrity of the plasma membrane was assessed using the hypoosmotic swelling test (HOST) with 100 μl of the sample diluted in 1.0 ml of a 100 mOsmol/l sucrose solution. The diluted samples were first incubated in a water bath at 37ºC for 30 min and were subsequently fixed with 500 μl of buffered formalin-saline, and 100 cells were evaluated using a phase contrast microscope (1000x; Olympus® BX 41). The percentage of cells reactive to HOST was calculated according to the method of Melo and Henry (1999).

### Fertility Trial

Eight cyclic mares of unknown fertility were used to test semen frozen in BotuCrio containing 12% of LDL. One insemination per estrous was performed in the tip of the uterine horn ipsilateral to the ovulatory follicle using a flexible pipette (Minitube do Brasil Ltda, Porto Alegre-RS, Brazil) after the occurrence of ovulation (maximum 6 h post-ovulation) using an average of 500 x 10^6^ progressively motile sperm. Pregnancy was diagnosed by ultrasound between 14 and 30 days after artificial insemination (AI).

### Statistical analysis

The first experimental design was in randomized blocks, considering the ejaculate as block. Descriptives analysis were performed. Kolmogorov-Smirnov test were performed for testing normality. The results were submitted to ANOVA and the averages obtained were compared by the Tukey test with probability level of 5%. The evaluated variables that did not have a normal distribution were analyzed by the Friedman test. For analysis it was used the statistical package of SAS® (Statistical Analysis System, SAS Institute Inc, Cary, NC, USA).

The second experimental design was a mixed-design analysis of variance model (split-plot ANOVA) considering animal as block, and observations time as subparcel. The variables were tested for normality test of Kolmogorov-Smirnov and those that do not answered the premise of normal distribution suffered radicial transformation (square root). Interaction between time and extender was not significant for all variables studied. The averages obtained were compared by the Tukey test with probability level of 5%. The variable Hyperactivity was analyzed by a non-parametric test of Wilcoxon. For analysis it was used the statistical package of SAS® (Statistical Analysis System, SAS Institute Inc, Cary, NC, USA).

## Results

### Experiment 1

There was an accentuated decrease of sperm quality between the fresh semen and the thawed one. The BotuCrio® extender was superior in preserving the motility, VCL, VSL, VAP, FAST, MED, LIN, STR and BCF when compared to the Merk-egg yolk and INRA 82 modified (P < 0.05). The use of BotuCrio® extender has resulted in a high percentage of hyperactive sperm when compared to other extenders (P < 0.05). For the sperm characteristics of SLOW and ALH, it was noticed that the BotuCrio® extender has preserved in a similar way to Merk-egg yolk modified and superior to the INRA 82 modified (P < 0.05; Tab. 1).

**Table 1:** Post-thaw sperm motion parameters of cryopreserved semen in three different extenders.

Parameters	BotuCrio®	Merk-egg yolk modified	INRA 82 modified
TM (%)	71.7 ± 8.8^a^	29.5 ± 17.1^b^	12.4 ± 6.3^c^
PM (%)	35.2 ± 18.1^a^	1.4 ± 1.1^b^	0.6 ± 0.6^bc^
VCL (µm/s)	64.9 ± 15.4^a^	27.4 ± 5.2^b^	24.7 ± 2.8^bc^
VSL (µm/s)	37.9 ± 11.7^a^	7.9 ± 2.7^b^	8.0 ± 2.2^bc^
VAP (µm/s)	45.1 ± 13.6^a^	13.7 ± 3.6^b^	13.4 ± 1.8^bc^
FAST (%)	25.4 ± 17.9^a^	1.1 ± 1.1^b^	0.3 ± 0.2^bc^
MED (%)	18.4 ± 8.8^a^	3.6 ± 3.1^b^	1.0 ± 0.9^c^
SLOW (%)	27.9 ± 9.4^a^	24.7 ± 13.4^a^	11.1 ± 5.5^b^
LIN (%)	57.5 ± 5.0^a^	28.2 ± 4.5^b^	34.5 ± 4.8^b^
STR (%)	84.1 ± 3.4^a^	56.5 ± 4.8^c^	64.0 ± 7.0^b^
ALH (µm/s)	3.3 ± 0.3^a^	3.0 ± 1.0^ab^	2.4 ± 0.8^b^
BCF (Hz)	11.8 ± 1.9^a^	6.5 ± 3.4^b^	7.0 ± 2.7^b^
Hyperactive (%)	25.8 ± 14.5^a^	3.2 ± 3.5^b^	0.8 ± 0.7^c^

a,b,c Average with different superscripts differ within the line (P < 0.05). X ± SD. TM, Total Motility; PM, Progressive Motility; VCL, Curvilinear Velocity; VSL, Straight Linear Velocity; VAP, Average Path Velocity; MED, Medium; LIN, Linearity; STR, Straightness; ALH, Amplitude of Lateral Head Displacement and BCF, Tail Beat Frequency. Merk-egg yolk and INRA 82 modified by the inclusion of acetamide, methylcellulose and trehalose in substitution of glycerol.

It was possible to verify accentuated decrease in motility after two hours of incubation at 37ºC regardless of extender tested (P < 0.05). The samples that showed acceptable progressive sperm motility after thawing reduced to less than 20% after incubation (P < 0.05; Tab. 2).

**Table 2:** Sperm motion parameters from equine sperm frozen/thawed in BotuCrio^®^, Merk-egg yolk and INRA 82 (both containing acetamide, methylcellulose and trehalose in substitution of glycerol) evaluated by optic microscopic during 2h of incubation at 37^o^C.

	Period of incubation		
	0h	1h	2h
Extender	TM (%)		
BotuCrio^®^	52.1 ± 16.5^aA^	40.3 ± 26.7^b^	21.8 ± 20.6^c^
Merk-egg yolk modified	37.4 ± 12.2^aAB^	21.5 ± 12.6^b^	11.5 ± 10.0^c^
INRA 82 modified	27.2 ± 9.0^aB^	14.4 ± 6.5^b^	4.2 ± 2.9^c^
Extender	PM (%)		
BotuCrio^®^	46.8 ± 17.0^aA^	36.0 ± 24.9^b^	18.8 ± 18.4^b^
Merk-egg yolk modified	30.0 ± 13.2^aA^	14.2 ± 12.8^b^	6.3 ± 8.1^c^
INRA 82 modified	14.5 ± 6.0^aB^	6.6 ± 4.4^b^	2.2 ± 0.6^c^

a,b,c Within a line, means without a common superscript differed (P < 0.05).^A,B^ Within a column, means without a common superscript differed (P < 0.05). X ± SD. TM, Total Motility; PM, Progressive Motility.

Structural integrity (ranged from 17.5% to 21.7%) and the plasma membrane functional integrity (ranged from 9.0% to 15.1% from reactive to the HOST) did not differ among cryopreserved samples in different extenders after thawing (P > 0.05).

### Experiment 2

There was a decrease of sperms quality between the fresh semen and thawed one. It was noticed that the progressive motility, FAST, VCL, VSL, VAP, LIN, STR, the percentage of hyperactive and functionally intact evaluated by the HOST was greater in the frozen semen with BotuCrio® egg yolk compared to the same extender containing 12% of LDL (P < 0.05; Tab. 3).

**Table 3:** Post-thaw sperm motion parameters and sperm viability of cryopreserved semen in BotuCrio® egg yolk and BotuCrio LDL extenders.

Parameters	BotuCrio® Egg yolk	BotuCrio LDL
TM (%)	37.5 ± 14.3	34.0 ± 15.3
PM (%)	10.1 ± 7.8^a^	4.5 ± 3.6^b^
VCL (µm/s)	36.0 ± 6.6^a^	28.6 ± 4.2^b^
VSL (µm/s)	18.3 ± 6.0^a^	13.0 ± 3.4^b^
VAP (µm/s)	23.3± 5.9^a^	18.1 ± 3.0^b^
FAST (%)	2.1 ± 1.9^a^	0.7 ± 0.78^b^
MED (%)	9.9 ± 7.1	5.7 ± 3.7
SLOW (%)	25.4 ± 7.2	27.6 ± 11.6
LIN (%)	50.0 ± 11.3^a^	45.0 ± 6.7^b^
STR (%)	76.9 ± 8.3^a^	70.9 ± 7.3^b^
ALH (µm/s)	2.7 ± 0.5	2.7 ± 0.4
BCF (Hz)	10.4 ± 1.4	9.4 ± 2.9
Hyperactive (%)	1.6 ± 1.7^a^	0.6 ± 0.7^b^
CFDA + (%)	50.1 ± 7.9	48.1 ± 11.1
HOST +(%)	32.4 ± 8.0^a^	18.2 ± 6.7^b^

a,b Average with different superscripts differ within the line (P < 0.05). X ± SD. TM, Total Motility; PM, Progressive Motility; VCL, Curvilinear Velocity; VSL, Straight Linear Velocity; VAP, Average Path Velocity; MED, Medium; LIN, Linearity; STR, Straightness; ALH, Amplitude of Lateral Head Displacement and BCF, Tail Beat Frequency. CFDA +, sperms with intact structural membrane stained by CFDA. HOST, reactive sperms to hypoosmotic swelling test.

It was noticed a decrease in total and progressive motility during incubation at 37ºC (P < 0.05; Tab. 4). The BotuCrio® extender containing egg yolk or LDL have preserved in a similar manner the total motility after thawing and after one and two hours of incubation at 37^o^C (P > 0.05). The progressive motility of cryopreserved sperm in the two tested extenders decreased after 1 h of incubation (P < 0.05), but remained with similar values until the second hour of evaluation (P > 0.05). However, the progressive motility was superior to the sperm frozen in BotuCrio® with egg yolk than in BotuCrio containing 12% of LDL in all evaluation moments during the incubation period (P < 0.05). At the end of the two hours of incubation, the motility was less than 20%.

**Table 4:** Kinetic parameters from equine sperm frozen/thawed in BotuCrio^®^ extender containing whole egg yolk or LDL evaluated by SCA® during 2h of incubation at 37^o^C.

	Period of incubation		
	0h	1h	2h
Extender	TM (%)		
BotuCrio^®^ Egg yolk	37.5 ± 14.3^a^	27.4 ± 19.2^b^	17.8 ± 14.9^c^
BotuCrio LDL	34.0 ± 15.3^a^	19.5 ±7.7^b^	10.7 ± 5.1^c^
Extender	PM (%)		
BotuCrio^®^ Egg yolk	10.1 ± 7.8^aA^	4.3 ± 5.4^bA^	2.7 ± 5.8^bA^
BotuCrio LDL	4.5 ± 3.6^aB^	1.4 ± 1.5^bB^	0.2 ± 0.3^bB^

a,b,c Within a line, means without a common superscript differed (P< 0.05). ^A,B^ Within a column, means without a common superscript differed (P< 0.05). X ± SD. TM, Total Motility; PM, Progressive Motility.

The fertility rate of eight mares inseminated with frozen semen in BotuCrio LDL was 37.5% (3/8).

## Discussion

The BotuCrio® extender containing methylformamide and glycerol was superior to preserve equine semen during cryopreservation than the extenders Merk-egg yolk and INRA 82 modified with the addition of acetamide, methyl cellulose and trehalose in substitution of glycerol. Other researchers have also reported this superiority of BotuCrio® on equine semen frozen with extenders containing glycerol as its main cryoprotectant agent (Terraciano *et al*., 2008; Candeias *et al*., 2012; Costa et al., 2014; Ramires Neto *et al*., 2014), especially to freeze semen from “bad freezer” stallions (Alvarenga *et al*., 2003; Squires *et al*., 2004; Ramires Neto *et al*., 2014) as were considered the majority of Mangalarga Marchador stallions breed (Alvarenga *et al*., 2003).

The cryoprotective capacity of Merk-egg yolk extender containing acetamide, methyl cellulose and trehalose on the viability of the equine sperms had been described previously (Henry *et al*., 2002; Snoeck *et al*., 2007; 2012). However, this cryoprotectant association had not yet been studied in INRA 82 extender. Despite the results obtained previously, it can be noticed that the use of the acetamide, methyl cellulose and trehalose in Merk-egg yolk extender and INRA 82 did not reach the cryoprotective capacity desired when compared to the BotuCrio®. It can be inferred that the composition of the BotuCrio® extender is superior to the composition of the Merk and INRA 82, given the combination of intra and extracellular cryoprotectant substances that can play an important role in sperms preservation at cooling and freezing process (Snoeck *et al*., 2007; Hoffman *et al*., 2011; Ramires Neto *et al*., 2014). Or even that the association between methylformamide and glycerol offers a better cryoprotection to the equine sperm as it has already been described by Rossi *et al*. (2003) and Melo *et al*. (2007).

Graham (2000) observed that the percentage of motile sperm after thawing was much less for semen frozen in skim milk egg yolk extender containing either acetamide or methyl acetamide when compared with methylformamide and dimethylformamide corroborating with the results obtained here. Alvarenga et al. (2005) and Melo *et al*. (2007) suggested that the relative lesser viscosity and low molecular weight of the amides, including methylformamide, likely favored an enhanced permeability of these compounds into the plasma membrane, resulting in a lesser osmotic damage to stallion sperm. This cryoprotectant agent property can explain the reason for the methylformamide in association with glycerol exercises superior cryoprotective effect to acetamide associated with metilcelulose and trehalose in tested extenders.

It was known that cryoprotectants which contains in its formulation the methyl or amide group are more effective in sperm cryopreservation (Hanada and Nagase, 1980; Dalimata and Graham 1997; Squires *et al*., 2004; Melo *et al*., 2007), however, other characteristics as well as low molecular weight, high permeability and low toxicity are needed to make a cryoagent effective in cell freezing (Hanada and Nagase, 1980). It was noticed that for equine sperm frozen using the BotuCrio®, the inclusion of low percentage of glycerol to the extender containing higher amount of methylformamide among other components assured important characteristics to be considered a better extender.

It has already been reported that the fraction that gives protection to the sperm against cold shock comes from low-density lipoproteins extracted from egg yolk (Moussa *et al*., 2002). Also, it has been shown in bovine that LDL protect sperm membranes by associating with seminal plasma proteins (Manjunath *et al*., 2002) preventing them from promoting cholesterol efflux of the membrane, and thereby triggering capacitation (Bergeron *et al*., 2004) which is unwanted during cryopreservation. Extenders containing LDL remained the sperm viability of many domestic mammal’s species in a similar manner or in a higher manner than egg yolk (Moussa *et al*., 2002; Amirat *et al*., 2004; Juliani *et al*., 2004; Jiang *et al*., 2007; Varela Júnior *et al*., 2009; Moustacas *et al*., 2011; Moreno *et al*., 2013; Neves *et al*., 2014; Silva *et al*., 2014).

In BotuCrio® the replacement of egg yolk for 12% of LDL has not resulted in an increase of mobility and movement after freezing and thawing, probably due to the extender property after its preparation. The extender containing LDL was produced using all components of the commercial extender without the egg yolk. It is worth mentioning that the BotuCrio containing LDL showed many particles in suspension which may have compromised some motion parameters evaluated by CASA and still demanded the elimination of granules by the cleanliness of the captured images, bearing in mind that the software recorded these particles as slow or stopped sperms. The frozen samples in BotuCrio® egg yolk showed perfect viewing by CASA.

In our laboratory, the use of LDL in extenders formulation, without detergent, produced either in liquid form, lyophilized or using pure LDL to be added to the medium at the time of sperm cryopreservation, all preserved at -20ºC or -80ºC until the use, did not result in the granules formation or particles in suspension (Snoeck *et al*., 2017). It is possible to assign that the poor result of BotuCrio containing LDL may be due to the percentage of LDL used or some negative interaction between LDL and other components of BotuCrio®, including the detergent used to dilute the egg yolk fats. It has been previously reported the presence of granules in extenders containing Equex® as a detergent (Bencharif *et al*., 2010). Another factor that may have contributed to the formation of these particles in suspension was the process of LDL inclusion to the extender. Our experience with the production of LDL and addition in cooling and freezing extenders of semen was always after the extraction or after a single thawing when the LDL was stored separately. In experiments with donkey, the LDL was added to the extender after extraction and then frozen until the semen cooling process (Melo *et al*., 2012).

Data on fertility with the use of equine frozen semen in BotuCrio extender containing LDL were not found. Despite the low number of inseminated mares, the fertility rate obtained was close to that found by other researchers who used only one insemination post-ovulation and semen frozen in extenders containing cryoprotectants from amides group (Oliveira *et al*., 2013; Avanzi *et al*., 2015). Fertility rates between 40 and 82.3% were reported after the use of two inseminations using an extender containing 4% of methylformamide and 1% glycerol (Melo *et al*., 2007). However, it is worth mentioning that the fertility rate depends on a number of factors such as freezing protocol, type of extender and cryoprotectants used, method and protocol of AI, sperm concentration of insemination dose, sensitivity of sperm from the donor to the process of cryopreservation among others.

Based on the results of this study, we can conclude that BotuCrio^®^ extender protected Mangalarga Marchador stallion sperm from cryodamage better than Merk-egg yolk and INRA 82 modified by the inclusion of acetamide, methyl cellulose and trehalose used as a replacement for glycerol. Analysis of the motility and structural integrity of the membrane associated with fertility result shows that the LDL could replace egg yolk in BotuCrio extender. Although studies on different concentrations, form of inclusion and preparation of extender containing LDL are needed to make the product available for commercial use.

## References

[B001] Alvarenga MA, Graham JK, Keith SL, Landim-Alvarenga FC, Squires EL (2000). Alternative cryoprotectors for freezing stallion spermatozoa.

[B002] Alvarenga MA, Leão KM, Papa FO, Graham Jk (2002). Improvement of stallion semen post-thaw motility with the utilization of dimethyl-formamide as cryoprotector. Theriogenology.

[B003] Alvarenga MA, Leão KM, Papa FO, Landim-Alvarenga FC, Medeiros ASL, Gomes GM (2003). The use of alternative cryoprotectors for freezing stallion semen.

[B004] Alvarenga MA, Papa FO, Landim-Alvarenga FC, Medeiros ASL (2005). Amides as cryoprotectans for freezing stallion semen: a review. Anim Reprod Sci.

[B005] Álvarez C, Gil L, González N, Olaciregui M, Luño V (2014). Equine sperm post-thaw evaluation after the addition of different cryoprotectants added to INRA 96® extender. Cryobiology.

[B006] Amirat L, Tainturier D, Jeanneau L, Thorin C, Gérard O, Courtens JL, Anton M (2004). Bull semen in vitro fertility after cryopreservation using egg yolk LDL: a comparison with Optidyl®, a commercial egg yolk extender. Theriogenology.

[B007] Avanzi BR, Ramos RS, Araujo GHM, Fioratii EG, Trinca LA, Dell’Aqua Jr JA, Melo E, Oña CM, Zahn FS, Martin I, Alvarenga MA, Papa FO (2015). Fixed-time insemination with frozen semen in mares: is it suitable for poorly fertile stallions?. Theriogenology.

[B008] Bergeron A, Crête MH, Brindle Y, Manjunath P (2004). Low-density lipoprotein fraction from hen’s egg yolk decreases the binding of the major proteins of bovine seminal plasma to sperm and prevents lipid efflux from the sperm membrane. Biol Reprod.

[B009] Bencharif D, Amirat-Briand L, Garand A, Anton M, Schmitt E, Desherces S, Delhomme G, Langlois ML, Barrière P, Destrumelle S, Vera-Munoz O, Tainturier D (2010). Freezing canine sperm: Comparison of semen extenders containing Equex® and LDL (Low Density Lipoproteins). Anim Reprod Sci.

[B010] Candeias ML, Alvarenga MA, Carmo MT, Ferreira HN, Maior MRS, Torres Filho RA, Rodrigues ALR, Brandão FZ (2012). Semen cryopreservation protocols of Mangalarga Marchador stallions. R Bras Zootec.

[B011] CBRA (2013). Manual for Andrological Examination and Evaluation of Animal Semen.

[B012] Costa DNM, Silva DAM, Boakari YL, Ferreira SB, Branco MAC, Souza JAT (2014). Efficiency of tris extenders and Botu-crio® on seminal parameters in stallion breeds Quarter Horse and Mangalarga Marchador. Cienc Anim Bras.

[B013] Dalimata AM, Graham J K (1997). Cryopresevation of rabbit spermatozoa using acetamide in combination with trehalose and methyl cellulose. Theriogenology.

[B014] Gibb Z, Morris LHA, Maxwell WMC, Grupen CG (2013). Dimethyl formamide improves the postthaw characteristics of sex-sorted and nonsorted stallion sperm. Theriogenology.

[B015] Gomes GM, Papa F O, Jacob JCF, Macedo LP, Leão KM, Machado MS, Alvarenga MA (2002). Improvement of frozen-thawed spermatic parameters with utilization of MP-50 extender to Mangalarga Machador stallions. Rev Bras Reprod Anim.

[B016] Graham JK (2000). Evaluation of alternative cryoprotectants for preserving stallion spermatozoa.

[B017] Hanada A, Nagase H (1980). Cryoprotective effects of some amides on rabbit spermatozoa. J Reprod Fert.

[B018] Harrison RAP, Vickers SE (1990). Use of fluorescent probes to assess membrane integrity in mammalian spermatozoa. J Reprod Fertil.

[B019] Henry M, Snoeck PPN, Cottorello ACP (2002). Post-thaw spermatozoa plasma membrane integrity and motility of stallion semen frozen with different cryoprotectants. Theriogenology.

[B020] Hoffmann N, Oldenhof H, Morandini C, Rohn K, Sieme H (2011). Optimal concentrations of cryoprotective agents for semen from stallions that are classified ‘good’ or ‘poor’ for freezing. Anim Reprod Sci.

[B021] Jiang ZL, Li QW, Li WY, Hu JH, Zhao HW, Zhang SS (2007). Effect of low density lipoprotein on DNA integrity of freezing-thawing boar sperm by neutral comet assay. Anim Reprod Sci.

[B022] Juliani G, Henry M, Melo MIV (2004). Freezing of equine semen in extenders with low density lipoproteins.

[B023] Magistrini M, Couty I, Palmer E (1992). Factors influencing stallion sperm survival. Acta Vet Scand Suppl.

[B024] Manjunath P, Nauc V, Bergeron A, Ménard M (2002). Major proteins of bovine seminal plasma bind to the low-density lipoprotein fraction of hen’s egg yolk. Biol Reprod.

[B025] Martin JC, Klug E, Günzel AR (1979). Centrifugation of stallion semen and its storage in large volumes straws. J Reprod Fertil Suppl.

[B026] Medeiros ASL, Gomes GM, Carmo MT, Papa FO, Alvarenga MA (2002). Cryopreservation of stallion using different amides. Theriogenology.

[B027] Melo CM, Zahn FS, Martin I, Orlandi C, Dell’Aqua Jr JA, Alvarenga MA, Papa FO (2007). Influence of semen storage and cryoprotectant on post-thaw viability and fertility of stallion spermatozoa. J Equine Vet Sci.

[B028] Melo MIV, Henry M (1999). Hypoosmotic test for the evaluation of equine semen. Arq Bras Med Vet Zootec.

[B029] Melo MIV, Resende YF, Rezende EP, Snoeck PPN, Gomes DML, Carvalho TTC, Silva AVD, Neves MM, Bastos DG, Henry MRJM (2012). Low density lipoproteins and egg yolk to preserve cooled donkey semen. Rev Bras Med Vet.

[B030] Moreno D, Bencharif D, Amirat-Briand L, Neira A, Destrumelle S, Tainturier D (2013). Preliminary results: the advantages of low-density lipoproteins for the cryopreservation of equine semen. J Equine Vet Sci.

[B031] Moussa M, Martinet V, Trimeche A, Tainturier D, Anton M (2002). Low density lipoproteins extracted from hen egg yolk by an easy method: cryoprotective effect on frozen-thawed bull semen. Theriogenology.

[B032] Moustacas VS, Zaffalon FG, Lagares MA, Loaiza-Eccheverri AM, Varago FC, Neves MM, Heneine LGD, Arruda RP, Henry M (2011). Natural, but not lyophilized, low density lypoproteins were an acceptable alternative to egg yolk for cryopreservation of ram. Theriogenology.

[B033] Neves MM, Heneine LGD, Henry M (2014). Cryoprotection effectiveness of low concentrations of natural and lyophilized LDL (low density lipoproteins) on canine spermatozoa. Arq Bras Med Vet Zootec.

[B034] Oliveira RA, Rubin MIB, Silva CAM (2013). Pregnancy rates using frozen semen of Criollo stallions with glycerol or dimethylformamid as cryoprotectants. Cienc Anim Bras.

[B035] Papa FO, Zahn FS, Dell’Aqua Jr JA, Alvarenga MA (2002). Using the MP 50 extender for cryopreservation of equine semen. Rev Bras Reprod Anim.

[B036] Pillet E, Batellier F, Duchamp G, Furstoss V, Vern YLE, Kerboeuf D, Vidament M, Renseigné N (2008). Freezing stallion semen in INRA96® based extender improves fertility rates in comparison with INRA82. Dairy Sci Technol.

[B037] Pillet E, Duchamp G, Batellier F, Beaumal V, Anton M, Desherces S, Schmitt E, Magistrini M (2011). Egg yolk plasma can replace egg yolk in stallion freezing extenders. Theriogenology.

[B038] Ramires Neto C, Monteiro GA, Sancler-Silva YFR, Papa P, Guasti PN, Resende HL, Papa FO, Dell’Áqua Jr JA, Alvarenga MA (2014). Comparison of different freezing extenders for semen cryopreservation from stallions with poor and good semen freezability. J Equine Vet Sci.

[B039] Rossi TC, Papa FO, Santos TB, Macedo LP, Alvarenga MA, Melo CM, Dell’Aqua Junior JA (2003). Efeito da utilização de diferentes crioprotetores e suas associações no processo de congelação de sêmen eqüino com meio MP50. Rev Bras Reprod Anim.

[B040] Silva MC, Moura LCO, Melo MIV, Mambrini JVM, Neves MM, Henry MRJM, Snoeck PPN. (2014). Prolonged post cooling but not pre-cooling equilibrium lengh improves the viability of ram sperm cryopreserved in an extender containing low-density lipoproteins. Small Ruminant Res.

[B041] Snoeck PPN, Cottorello ACP, Henry M (2012). Viability and fertility of stallion semen frozen with ethylene glycol and acetamide as a cryogenic agent. Anim Reprod.

[B042] Snoeck PPN, Henry M, Melo MIV (2007). Effect of differents freezing extenders on post thaw equine spermatozoa viability. Arq Bras Med Vet Zootec.

[B043] Snoeck PPN, Moura LCO, Silva MC, Machado-Neves M, Melo MIV, Heneine LGD, Henry M (2017). Effect of storage conditions on the LDL effectiveness in ovine sperm cryopreservation. Cryobiology.

[B044] Squires EL, Keith SL, Graham JK (2004). Evaluation of alternative cryoprotectants for preserving stallion spermatozoa. Theriogenology.

[B045] Terraciano PB, Bustamante – Filho IC, Miquelito LV, Arlas TR, Castro F, Mattos RC, Passos EP, Oberst ER, Lima EOC (2008). Cryopreservation of equine spermatozoa comparing different freezing rates combined with commercial extenders: laboratorial analysis. Cienc Rural.

[B046] Varela Junior AS, Corcini CD, Ulguim RR, Alvarenga MVF, Bianchi I, Corrêa MN, Lucia Jr T, Deschamps JC (2009). Effect of low density lipoprotein on the quality of cryopreserved dog semen. Anim Reprod Sci.

[B047] Vidament M, Daire C, Yvon J M., Doligez P, Bruneau B, Magistrini M, Ecot P. (2002). Motility and fertility of stallion semen frozen with glycerol and/or dimethyl formamide. Theriogenology.

